# Molecular Characterization of Clinical Isolates of *Aeromonas* Species from Malaysia

**DOI:** 10.1371/journal.pone.0030205

**Published:** 2012-02-27

**Authors:** S. D. Puthucheary, Suat Moi Puah, Kek Heng Chua

**Affiliations:** 1 Department of Medical Microbiology, Faculty of Medicine, University of Malaya, Kuala Lumpur, Malaysia; 2 Department of Molecular Medicine, Faculty of Medicine, University of Malaya, Kuala Lumpur, Malaysia; Monash University, Australia

## Abstract

**Background:**

*Aeromonas* species are common inhabitants of aquatic environments giving rise to infections in both fish and humans. Identification of aeromonads to the species level is problematic and complex due to their phenotypic and genotypic heterogeneity.

**Methodology/Principal Findings:**

*Aeromonas hydrophila* or *Aeromonas* sp were genetically re-identified using a combination of previously published methods targeting *GCAT*, 16S rDNA and *rpoD* genes. Characterization based on the genus specific *GCAT*-PCR showed that 94 (96%) of the 98 strains belonged to the genus *Aeromonas*. Considering the patterns obtained for the 94 isolates with the 16S rDNA-RFLP identification method, 3 clusters were recognised, i.e. *A*. *caviae* (61%), *A. hydrophila* (17%) and an unknown group (22%) with atypical RFLP restriction patterns. However, the phylogenetic tree constructed with the obtained *rpoD* sequences showed that 47 strains (50%) clustered with the sequence of the type strain of *A. aquariorum*, 18 (19%) with *A. caviae*, 16 (17%) with *A. hydrophila*, 12 (13%) with *A. veronii* and one strain (1%) with the type strain of *A. trota*. PCR investigation revealed the presence of 10 virulence genes in the 94 isolates as: *lip* (91%), *exu* (87%), *ela* (86%), *alt* (79%), *se*r (77%), *fla* (74%), *aer* (72%), *act* (43%), *aexT* (24%) and *ast* (23%).

**Conclusions/Significance:**

This study emphasizes the importance of using more than one method for the correct identification of *Aeromonas* strains. The sequences of the *rpoD* gene enabled the unambiguous identication of the 94 *Aeromonas* isolates in accordance with results of other recent studies. *Aeromonas aquariorum* showed to be the most prevalent species (50%) containing an important subset of virulence genes *lip/alt/ser/fla/aer*. Different combinations of the virulence genes present in the isolates indicate their probable role in the pathogenesis of *Aeromonas* infections.

## Introduction

Aeromonads are essentially ubiquitous in the microbial biosphere. They can be isolated from virtually every environmental niche where bacterial ecosystems exist. These include aquatic habitats, fish, foods, domesticated pets, invertebrate species, birds, ticks and insects, and natural soils, although extensive investigations on the latter subject are lacking. The vast panorama of environmental sources from which aeromonads can be encountered lends itself readily to constant exposure and interactions between the genus *Aeromonas* and humans [1], [2].

The genus *Aeromonas* consists of approximately 25 species and is classified into 2 main groups; the psycrophilic non-motile aeromonads infecting fish and reptiles and a larger group of motile mesophilic aeromonads which are responsible for and associated with a range of human diseases [2]. The exact incidence of *Aeromonas* infection on a global basis is unknown since many cases either go undetected or are not reported.

Aeromonads are responsible for a “cornucopia” of intestinal and extra intestinal diseases and syndromes, ranging from relatively mild illnesses such as acute gastroenteritis to life-threatening conditions, including septicemia, necrotizing fasciitis, and myonecrosis [1]. In Malaysia we have reported this organism giving rise to both intestinal as well as extra intestinal infections such as septicaemia, peritonitis, osteomyelitis and soft tissue infections [3].

The mechanism of pathogenesis is complex and unclear [1], [2]. All genes that encode for virulence associated factors that allow the pathogen to establish infection in the host are defined as virulence genes. Virulence of aeromonads is considered to be multifactorial including cytotonic heat-labile (*alt*) [4],and cytotonic heat-stable enterotoxins (*ast*) [5], cytotoxic heat-labile enterotoxin (*act*) [6], aerolysin (*aer*) [7], flagella A and flagella B (*fla*) [8], lipase (*lip*) [8], elastase (*ela*) [8], serine protease (*ser*) [9], ADP-ribosyltransferase toxin (*aexT*) [10], and DNases (*exu*) [11]. It is not clear whether there is a virulent subset of *Aeromonas* species prevalent in clinical isolates with the ability to cause human infections. Therefore, the detection of virulence genes in *Aeromonas* is essential in determining potential pathogenicity of the organism and subsequent possible targets for prevention of infection.

Members of the genus *Aeromonas* are not difficult to isolate from clinical specimens in the diagnostic laboratory, but are often misidentified as belonging to the genus *Vibrio* or *Plesiomonas* [1], [12], [13]. To avoid confusion with other genera a specific PCR probe for the genus *Aeromonas* targeting the glycerophospholipid-cholesterol acyltransferase (*GCAT*) gene was designed by Chacon et al. [12]. These authors demonstrated that this gene was present in practically all *Aeromonas* strains tested, including representatives of all species [12]. The detection of the *GCAT* gene by PCR enabled Beaz-Hidalgo et al. [13] to recognize that only 75.6% (90/119) of the phenotypically identified *Aeromonas* strains from diseased fish belonged to the genus. Identification of aeromonads to the species level is difficult and complex due to their phenotypic and genotypic heterogeneity [1], [13]–18. Commercial identification systems are also not useful for the identification of *Aeromonas* species [19], [20]. The use of molecular approaches has led to a more refined identification of *Aeromonas* species that has highlighted a number of discrepancies in biochemical identification of both environmental and clinical isolates [13], [17], [18].

Molecular techniques have been developed to overcome these problems of identification but one limitation of such techniques is that many of the DNA probes for *Aeromonas* have a very narrow spectrum allowing for the identification of only one species at a time [2]. 16S rDNA gene sequencing used for bacterial genus and species identification is straightforward and largely reliable. But difficulties can arise due to high sequence divergence in the 16S rDNA genes in different strains of the same species which can be up to 1.5% [15]. The presence of nucleotide polymorphism among the rrn operons of the 16S rDNA, i.e., microheterogeneities have produced unexpected or atypical restriction patterns making identification of species uncertain, which were then correctly identified using housekeeping *gyrB* and *rpoD* gene sequences [16]. Housekeeping gene *rpoD* provided unequivocal identification of *Aeromonas* species of ichthyopathological importance [13] and the glycerophospholipid-cholesterol acyltransferase (*GCAT*) gene was found to be present in practically all *Aeromonas* strains tested, including representatives of all species [12].

All the above studies had used each of the three i.e. 16S rDNA, the *GCAT* or the *rpoD* individually or in a combination of 2 for the identification of *Aeromonas* species. Therefore, the aim of our study was to identify and speciate clinical isolates of *Aeromonas* strains by using a combination gene analysis of *GCAT*, 16S rDNA and *rpoD*, and to detect the distribution of 10 known virulence genes in order to provide relevance, knowledge and understanding to the pathogenicity of *Aeromonas* infections.

## Materials and Methods

### Bacterial strains

A total of 98 clinical isolates of *Aeromonas* species obtained from patients at the University Hospital, University of Malaya (UM), Kuala Lumpur, were investigated in this study. Specimens included blood, pus, tissues and body fluids, urine, sputum and peritoneal dialysates. The University Hospital is a tertiary referral facility and most of the patients were admitted as in-patients. Twenty five of 89 (28%) patients were children with a mean age range of one month to 4 years, and the range in 64 (72%) adults was from 36 to 49 years. Five samples had insufficient demographic data (Table 1). The strains had been isolated on blood agar, desoxycholate citrate agar, thiosulphate citrate bile salts sucrose agar and identified at least to genus level by the API 20E system (bioMérieux, France), in a previous study [21] and cryopreserved in 20% glycerol at −80°C. Working cultures were maintained in Luria Bertani (LB) agar and broth.

**Table 1:** Characteristics of the 94 patients with *Aeromonas* isolates recovered from Malaysia.

			Disease spectrum		
	Primary bacteremia	Acute gastroenteritis	Peritoneal dialysate	*Soft tissue infection	Others
**No. of patients**	4	52	13	22	3
**Case**					
Children	1	18	2	4	0
Adult	3	31	11	16	3
Insufficient data	0	3	0	2	0
**Age, Mean (year)**					
Children	1 month	2	4	3	0
Adult	39	48	49	36	40
**Species distribution**					
*A.aquariorum*	0	27	6	13	2
*A.caviae*	1	11	3	2	0
*A.hydrophila*	2	4	4	6	0
*A.veronii*	1	9	0	1	1
*A.trota*	0	1	0	0	0

### Ethics Statement

These *Aeromonas* isolates were from sporadic cases seen at the University Hospital, University of Malaya from 1982 to 1990. Verbal consent was obtained from patients for blood as well as for other samples before collection and it was understood that these were for diagnostic and research purposes and this was sufficient at that material time. The *Aeromonas* isolates had been archived and retrieved previously for related studies [21], [22].

### Genomic DNA extraction and purification

The Bacterial Genomic DNA Isolation Kit (Norgen Biotek, Canada) was used for genomic DNA extraction according to the manufacturer's protocol. Briefly, the bacterial culture was pelleted, resuspended and the cells lysed with proteinase K. The released bacterial DNA was passed through a column and washed to remove impurities. The purified bacterial DNA was eluted into 100 µL of buffer and subjected to spectrophotometric measurement. The extracted DNA was stored at −20°C for further use.

### Molecular identification and typing

The primer pairs used for PCR amplification and sequencing of *rpoD* and the specific conditions for the investigation of *GCAT*, 16S rDNA and *rpoD* genes were as reported previously [7], [17], [23]. PCR [7] and PCR-RFLP [17] were carried out to detect the *GCAT* and 16S rDNA genes. Digestion of the amplified 16S rDNA product was carried out for 3 hours at 37°C using 2 U of *Alu*I (New Englands Biolabs, USA) and *Mbo*I (New England Biolabs, USA). These digested products were electrophoretically separated on 18% v/v PAGE at 160V for 5 hours. A fragment of approximately 816 bp of the *rpoD* gene was amplified and purified using the QIAquick Gel Extraction kit (Qiagen, Germany). The purified products of all the strains were then sent for sequencing (1^st^ Base Laboratories, Malaysia) and results compared in a BLAST homology search with *Aeromonas* gene sequences deposited in the GenBank database. A representative number of the sequences of each species was confirmed by *gyrB* direct sequencing [23].

### Phylogenetic data analysis

The nucleotide sequences of *rpoD* of the strains (GenBank accession numbers: JN686647-JN686741) were aligned and pairwise sequence identity matrix was calculated by the Bioedit program 7.0.9 [24]. A phylogenetic tree was constructed by the neighbor-joining method [25] using the MEGA 4 program [26] and genetic distances were computed by using Kimura's two-parameter model [27]. The reference gene sequences of the following strains were obtained from NCBI: *A. aquariorum* MDC47 (FJ936132.1), *A. hydrophila subsp. dhakensis* CECT 5744 (EF465510.1), *A. aquariorum* MDC318 (EU268461.1), *A. hydrophila* CIP107985 (DQ448290.1), *A. hydrophila* ATCC 7966 (FN773322.1), *A. caviae* CECT 838 (HQ442790.1), *A. enteropelogenes* CECT 4487 (EU303299.1), *A. veronii* CECT 4246 (HQ442829.1) and *Vibrio parahaemolyticus* ATCC 17802 (AY527393.1).

### Detection of virulence genes

The 94 isolates identified as *Aeromonas* species by the presence of *GCAT*, were subjected to direct PCR to detect the presence of 10 virulence genes i.e. *alt*, *ast*, *act*, *aer*, *fla*, *lip*, *ela*, *ser*, *aexT* and e*xu*, using primers and conditions as described earlier [4]–[11]. Statistical analysis was carried out for association of combination virulence genes by two-tailed Fisher's exact test.

## Results and Discussion

On the basis of the *GCAT* results, 4 of the 98 strains (4%) did not belong to the genus *Aeromonas* thereby corroborating earlier work by Chacon et al. [12]. These 4 isolates were subsequently confirmed as non-*Aeromonas* by *rpoD* sequencing that identified 2 as *Serratia plymuthica*, one as *Vibrio parahaemolyticus* and another as *Vibrio harveyi* (Table 2).

**Table 2:** Identification and speciation of 98 *Aeromonas* clinical isolates using *GCAT*, 16S rDNA-RFLP and *rpoD* genes.

Phenotypes	*GCAT* screening	16S rDNA-RFLP	*rpoD* sequencing
98 isolates of	94 *Aeromonas* spp.	16 *A. hydrophila*	16 *A. hydrophila*
*Aeromonas spp.*		57 *A.caviae*	18 *A.caviae*
			39 *A.aquariorum*
		21 Unknown	1 *A.trota*
			8 *A.aquariorum*
			12 *A.veronii*
	4 Non-*Aeromonas* spp.	Not done	1 *Vibrio parahaemolyticus*
			1 *Vibrio harveyi*
			2 *Serratia plymuthica*

**Figure 1:**
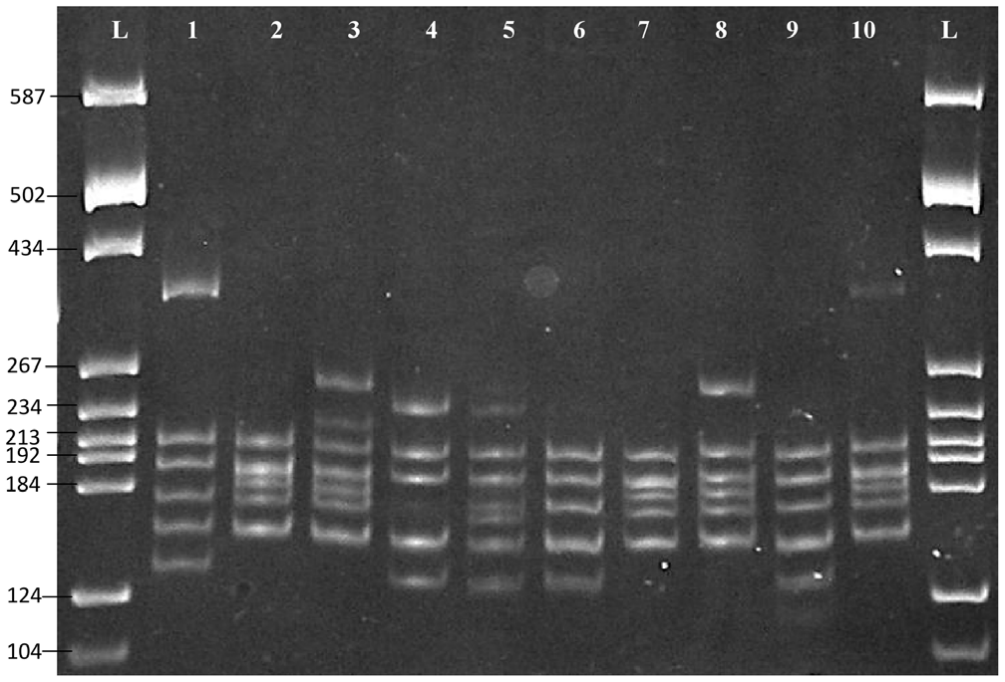
Polyacrylamide gel showing 16S rDNA-RFLP patterns (*Alu*I and *Mbo*I). L: pBR322 DNA/*BsuRI* marker (Fermentas, USA), Lane 1: typical pattern of *A. hydrophila* (JN 686656), Lane 2: typical pattern of *A. caviae* (JN 686668), Lane 3: atypical pattern of *A. trota* (JN 686649), Lanes 4–6: atypical pattern of *A. veronii* (JN 686665, JN 686691, JN 686739), Lanes 7–10: *A. aquariorum* (JN 686662, JN 686731, JN 686725, JN 686700).

The 94 *GCAT* positive isolates were subjected to 16S rDNA-RFLP and results showed that 73 isolates (78%) exhibited a common “typical” restriction pattern, i.e, 57 strains (61%) possessed the RFLP pattern of *A. caviae* and 16 strains (17%) that of *A. hydrophila*, and 21 strains (22%) had atypical patterns (Table 2, Figure 1). A common “typical” restriction pattern refers to the DNA fingerprints constituting a specific blueprint that can be used to identify a strain to the phylogenetic level of the species as described by Borrell et al. and Figueras et al. If the digested pattern differs from the “typical” blueprint, it is considered as an atypical RFLP pattern and this maybe expected if the digested sequence belongs to a new *Aeromonas* species, which had not been described in the last decade [17], [18].

Another possible explanation for the atypical pattern may be the differences present between strains of the same species, i.e. intra-species nucleotide diversity in the 16S rDNA genes in different strains of the same species. Sequencing of representative strains with atypical patterns showed that double sequencing signals (microheterogeneities) were present in the 16S rDNA gene, thus affecting definitive identification (data not shown). The degree of resolution obtained with 16S rDNA-RFLP was not sufficient to identify the species in the “atypical” pattern group, thus emphasizing the need for additional tests. In order to overcome this, additional investigations were undertaken for the conclusive identification of *Aeromonas* species. The use of housekeeping genes has been proposed to overcome this lack of accurate identification by 16S rDNA-RFLP [28].

The amplified products of housekeeping gene *rpoD* of all the 94 strains were sent for direct sequencing (1^st^ Base Laboratories, Malaysia). Concordance between 16S rDNA-RFLP assay and *rpoD* direct sequencing resulted in 16 isolates being identified as *A. hydrophila.* However, of the 57 strains showing the 16S rDNA-RFLP pattern of *A. caviae, rpoD* sequencing distinguished only 18 as *A. caviae* and 39 as *A. aquariorum* (Lanes 7–10, Figure 1). Our results concur with previous studies that 16S rDNA-RFLP pattern of *A. aquariorum* is very similar to that of *A. caviae*, making identification of species uncertain [29], [30]. Such a phenomenon may arise from the presence of nucleotide polymorphisms among the rrn operons of the 16S rRNA gene (so-called microheterogeneities) [16], [28].

The *rpoD* gene has proven to be an excellent molecular tool for inferring the taxonomy of *Aeromonas* and with the use of this gene, all our strains were unambiguously identified in agreement with Beaz-Hidalgo et al. [13] that *rpoD* helped improve the reliability of the phylogenies together with the 16S rDNA in environmental strains of *Aeromonas*. The unknown group of 21 isolates by RFLP, were identified by *rpoD* sequencing as follows: 12 as *A. veronii*, 8 as *A*. *aquariorum*, and one as *A. trota*. Several representative strains of each species were sequenced, using housekeeping gene *gyrB* which demonstrated similar discriminatory power as the *rpoD* gene sequence (data not shown), confirming the usefulness of this method for the identification of *Aeromonas* strains.

**Figure 2:**
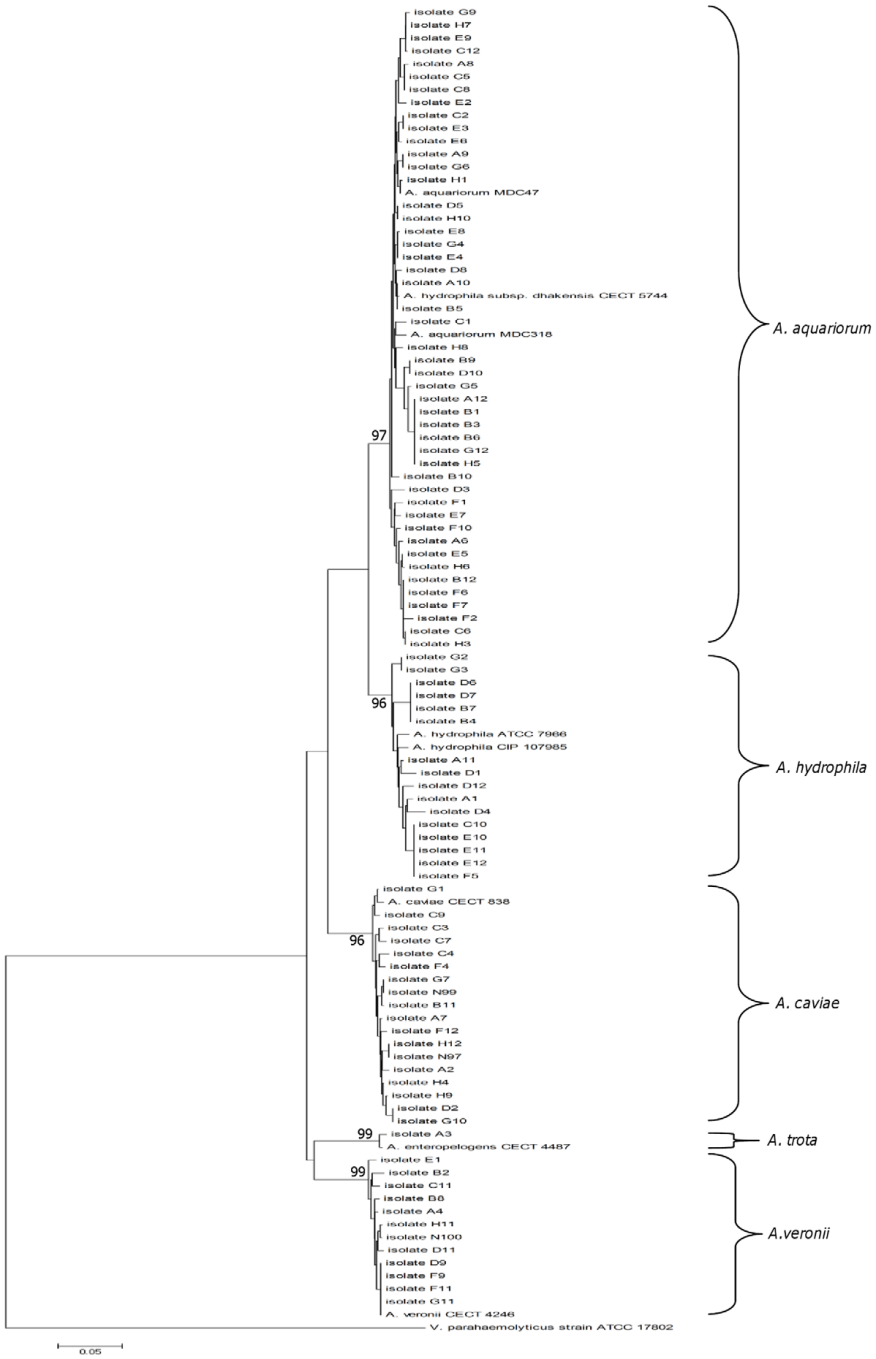
Phylogenetic relationship of the *rpoD* sequences between 94 *Aeromonas* isolates and 9 references strains using neighbor-joining method. Numbers next to nodes indicate percentage bootstrap values of 5000 replicates.

Based on the partial *rpoD* sequence alignment (461 bp), the intraspecies similarity for aeromonad isolates was 97.1–100% for *A. hydrophila* (n = 16), 96.9–100% for *A. aquariorum* (n = 47), and above 98% for both *A. caviae* (n = 18) and *A. veronii* (n = 12). In contrast, the sequence similarity between species diverged from 88.7% to 94.1%. A high sequence similarity of 94.1% was seen between *A. aquariorum* and *A. hydrophila*, indicating a close genetic relationship between these 2 species. The phylogenetic tree constructed by using *rpoD* gene sequences showed distinct clustering of species with high bootstrap values, ranging from 96% to 99%. (Figure 2), The derived neighbor-joining tree method based on Kimura 2-parameter model grouped all 94 strains into the following: 47 as *A. aquariorum*, 16 as *A*. *hydrophila*, 18 as *A. caviae* , 12 as *A. veronii*, and one as *A. trota* (Figure 2).

In the present study, *A. aquariorum* (50%) was the most prevalent among the clinical strains and is in accordance with other studies [29], [31]. It was isolated from cases of acute gastroenteritis, peritoneal dialysate and soft tissue infections (Table 1). It was the most prevalent in stool (n = 27, 57.4%) and 13 strains (27.7%) from pus and pus swabs, from osteomyelitis (n = 3), wounds (n = 3), hand injury (n = 2), cellulitis (n = 2), unknown source (n = 2), and abscess (n = 1) (Table 3). Besides isolation of *A. aquarorium* from ornamental fish and water from aquaria [32], this species has also been found in chironomid egg masses [33], indicating the diversity of its habitat, lending credence to the importance of *A. aquariorum* and its relevance in the clinical setting [29]. Another unexpected finding was the isolation and identification *A. trota* from a stool specimen. This is an ampicillin susceptible species and was from a 41-year-old male patient with severe gastroenteritis and watery diarrhoea with fever and vomiting. It is a unique species with very few reports and, further studies to characterize *A. trota* are essential for elucidating its pathogenesis and virulence.

**Table 3:** Source and distribution of 94 clinical isolates of *Aeromonas* species.

Site of isolation/infection	*A.aquariorum* (%)	*A.caviae* (%)	*A.hydrophila* (%)	*A.veronii* (%)	*A.trota* (%)	Total (%)
**Stool**	27 (57.4)	11 (61.1)	4 (25.0)	9 (75.0)	1 (100)	52 (55.3)
**Peritoneal dialysate**	5 (10.6)	4 (22.2)	4 (25.0)	0	0	13 (13.8)
**Blood**	0	1 (5.6)	2 (12.5)	1 (8.3)	0	4 (4.3)
**Pus/Pus swab** a	13 (27.7)	2 (11.1)	6 (37.5)	1 (8.3)	0	22 (23.4)
**Others** b	2 (4.3)	0	0	1 (8.3)	0	3 (3.2)
**Total**	**47 (100)**	**18 (100)**	**16 (100)**	**12 (100)**	**1 (100)**	**94 (100)**

a Pus/Pus swab from wounds , hand injury, cellulitis, abscess and unknown source.

b Two *A. aquariorum* from tracheal secretion and urine; one *A. veronii* from biliary tract secretion.

Harbourage of multiple virulence genes was common among the 94 *Aeromonas* isolates similar to previous reports [6]–[8], [34]–[37]. The two *A. aquariorum* isolates from stool and pus, and one *A. veronii* from stool, carried the full complement of the 10 virulence genes. The pus isolate of *A*. *aquariorum* was from a child of 4 years with hand injury. Of the 10 virulence genes the *lip* gene (91%) was the most prevalent found in 86 of the 94 isolates followed by *exu* (87%), *ela* (86%), *alt* (79%), *ser* (77%), *fla* (74%), *aer* (72%), *act* (43%), *aexT* (24%) and *ast* (23%) (Table 4). The gene encoding *lip* was the most prevalent regardless of source of isolation and it is tempting to hypothesize that *lip* gene might play an important role in *Aeromonas* infections. An earlier study reported that *A. hydrophila* with insertion mutants for the lipase gene reduced the lethal dose in mice and fish models [38]. Further studies on the lipase gene in non-*A. hydrophila* species may provide insights into the pathogenesis of *Aeromonas* infections.

**Table 4:** Distribution of virulence genes in 94 clinical isolates of *Aeromonas.*

Virulence genes	*A.aquariorum* (n = 47) (%)	*A.caviae* (n = 18) (%)	*A.hydrophila* (n = 16) (%)	*A.veronii* (n = 12) (%)	*A.trota* (n = 1) (%)	Total (n = 94) (%)
***aer***	41 (87)	4 (22)	10 (63)	12 (100)	1 (100)	68 (72)
***alt***	46 (98)	6 (33)	16 (100)	5 (42)	1 (100)	74 (79)
***ast***	4 (9)	0	16 (100)	1 (8)	1 (100)	22(23)
***act***	18 (38)	1 (6)	9 (56)	12 (100)	0	40 (43)
***fla***	41 (87)	13 (72)	11 (69)	4 (33)	1 (100)	70 (74)
***lip***	47 (100)	18 (100)	16 (100)	4 (33)	1 (100)	86 (91)
***ela***	47 (100)	18 (100)	15 (94)	1 (8)	0	81 (86)
***exu***	41 (87)	17 (94)	13 (81)	11 (92)	0	82 (87)
***ser***	42 (89)	3 (17)	16 (100)	10 (83)	1 (100)	72 (77)
***aexT***	15 (32)	0	4 (25)	4 (33)	0	23 (24)

The 5 most common virulence genes present in all the 5 species of *Aeromonas* were *lip*, *alt*, *ser*, *fla* and *aer* (Table 4) and combination analysis based on these 5 genes revealed 17 “virulence” patterns. Different species carried distinct sets of these 5 common virulence genes in combination, and this observation led us to hypothesize that each species had a distinct set of virulence genes, but a statistically significant (*p*<0.001) association was only seen with *A*. *aquariorum* with *lip/alt/ser/fla/aer*; *A. hydrophila* with *lip/alt/ser/fla*; *A. caviae* with *lip/fla* and *A. veronii with alt/ser/aer*. The most frequently isolated was *A*. *aquariorum* and we believe that this species containing a subset of virulence genes as mentioned above may be responsible for a wide range of infections, as the 47 isolates were from 11 different body sites. Despite its clinical importance, little is known about its interactions with the host and future *in vitro* and *in vivo* work may give us clues to its virulence and pathogenicity.

In the present work 98 clinical isolates phenotypically classified as *Aeromonas* species were genetically re-identified using *GCAT* gene, 16S rDNA-RFLP and sequencing of the *rpoD* gene. Our results suggest that the use of 2 genes, *GCAT* and *rpoD* unambiguously identified 94 *Aeromonas* species according to recent taxonomical classification. In addition, the majority of isolates recovered from different clinical sources carried multiple virulence genes and these findings support the notion that different subsets of virulence genes exist in various *Aeromonas* species.
